# P-1160. Surge of Pediatric *Streptococcus pneumoniae* Infections at a Tertiary Healthcare Center after Introduction of 15 and 20 Valent Pneumococcal Conjugate Vaccines: Early Evidence of Serotype Replacement?

**DOI:** 10.1093/ofid/ofae631.1346

**Published:** 2025-01-29

**Authors:** Kyle Burkhart, Ujwal Aluru, Ali Abolhassani, Bradley Topping, Ediza M Giraldez, Laura L Hampton, Hasan Samra, Srilatha Neshangi, Ingrid Camelo

**Affiliations:** Medical College of Georgia at Augusta University, Augusta, Georgia; Medical College of Georgia at Augusta University, Augusta, Georgia; Medical College of Georgia at Augusta University, Augusta, Georgia; WMCG, Augusta, Georgia; MCG Wellstar Augusta, Augusta, Georgia; Medical College of Georgia at Augusta University, Augusta, Georgia; Augusta University, Augusta, Georgia; Augusta University Medical Center, Augusta, Georgia; Children's Hospital of Georgia, Augusta, Georgia

## Abstract

**Background:**

Routine pneumococcal vaccination has contributed to the overall decrease in pediatric cases of invasive pneumococcal disease (IPD). However, there has been an increase in non-vaccine serotypes (NVT) causing IPD in North America, Europe, and Australia. We describe hospital admissions of Streptococcus pneumoniae-related illness at a tertiary health care center from 2022-2024.

**Methods:**

Retrospective chart review was conducted of IPD and non-IPD cases admitted to the Children’s Hospital of Georgia from April 2022-April 2023 and April 2023-April 2024. We used the Centers for Disease Control and Prevention (CDC) definition of IPD. IPD and non-IPD disease cases were analyzed for site of infection, concomitant viral infections, vaccination status, Pediatric Intensive Care Unit (PICU) admission, and death.

**Results:**

From 2023-2024, there were 12 cases of IPD, 8 had bacteremia, 3 had meningitis and bacteremia, and 1 had meningitis compared to only 3 IPD cases in 2022-2023, all with bacteremia. 7 of the 12 cases (58%) of IPD cases in 2023-2024 were fully PCV13 vaccinated compared to 1 of the 3 cases (33%) in 2022-2023. Only RSV and rhino/enterovirus were found concomitantly. 7 patients required PICU admission in 2023-2024, 1 on 2022-2023. 1 patient died during 2023-2024. 2 cases had serotyping during 2023-2024; one had serotype 38 and the other had serotype 10A. PCV20 includes serotype 10A. None of the above serotypes are included in the PCV 13 or PCV 15 vaccines.

From 2023-2024, there were 9 cases of non-IPD infection compared to 13 cases in 2022-2023, all of which had pneumonia as the site of infection. Only 5 cases were fully immunized in 2023-2024 while 8 were vaccinated in 2022-2023. Concurrent viruses included parainfluenza, human metapneumovirus, SARS-CoV-2, coronavirus OC43, adenovirus, and rhinovirus. 4 patients required PICU admission in 2023-2024 and 5 in 2022-2023. 1 case died during 2023-2024, and 1 died during 2022-2023.

**Conclusion:**

The recent surge in IPD cases at our institution could be related to early serotype replacement following vaccine-expanded serotypes since more children with IPD were immunized during the 2023-2024 period. Increased surveillance and serotyping will guide decisions about increased IPD infections. Immunization efforts need to be reinforced in our community.

**Disclosures:**

**All Authors**: No reported disclosures

